# Adrenal tracer uptake by ^18^F-FDOPA PET/CT in patients with pheochromocytoma and controls

**DOI:** 10.1007/s00259-019-04332-5

**Published:** 2019-04-22

**Authors:** Walter Noordzij, Andor W. J. M. Glaudemans, Mirte Schaafsma, Anouk N. A. van der Horst-Schrivers, Riemer H. J. A. Slart, André P. van Beek, Michiel N. Kerstens

**Affiliations:** 1 Medical Imaging Center, Department of Nuclear Medicine & Molecular Imaging, University of Groningen, University Medical Center Groningen, EB 50, P.O. Box 30.001, 9700 RB Groningen, The Netherlands; 2 Department of Endocrinology, University of Groningen, University Medical Center Groningen, Groningen, The Netherlands; 3 TechMed Centre, Department of BMPI, University of Twente, Enschede, The Netherlands

**Keywords:** ^18^F-FDOPA, Pheochromocytoma, Adrenal glands, Normative data

## Abstract

**Context:**

^18^F-FDOPA PET/CT accurately localizes pheochromocytoma in patients with an established biochemical diagnosis. However, cut-off ^18^F-FDOPA levels of standardized uptake values (SUV_max_) for both normal adrenal glands and pheochromocytoma are lacking.

**Objective:**

Objectives of this study were to determine (1) reference maximum standardized uptake values (SUVmax) for normal adrenal ^18^F-DOPA tracer uptake and (2) the optimal diagnostic approach for pheochromocytoma localization by using ^18^F-DOPA SUVmax across a series of cut-off points: the affected adrenal gland (inter-individual analysis), the difference in SUVmax between the affected adrenal gland and the contralateral normal adrenal gland (intra-individual analysis), or a combination of these two.

**Patients and methods:**

All patients with histologically confirmed pheochromocytoma diagnosed at our center between November 2009 and December 2017 were retrospectively analysed. Only those patients who underwent an ^18^F-FDOPA PET/CT-scan for localization purposes before adrenalectomy were included for further analysis. The control group consisted of patients who underwent ^18^F-FDOPA PET/CT for other indications and who had no genetic susceptibility for developing a pheochromocytoma. SUV_max_ of the volume of interest surrounding the adrenal glands was determined on EARL reconstructed images. Receiver operating characteristic (ROC) analysis was performed for adrenal gland SUV_max_ and intra-individual difference in SUV_max_ between affected and normal adrenal gland. In addition, binary logistic regression was performed for ROC analysis of the combined parameters.

**Results:**

In total, 47 histologically confirmed pheochromocytomas were diagnosed in 45 patients, and 245 disease control patients were identified. In the control group, no statistical differences between the SUV_max_ of left and right adrenal glands were observed, and uptake values in both adrenal glands correlated significantly with each other (r = 0.865, *p* < 0.001). Median (range) adrenal gland SUV_max_ in pheochromocytomas and in the control group was 12 (2.6–50) and 2.9 (1.1–6.6), respectively (*p* < 0.001). ROC analysis revealed 93% sensitivity and 85% specificity at an SUV_max_ cut-off value of 4.1 (area under the curve (AUC) = 0.951), and 93% sensitivity and 96% specificity at an intra-individual SUV_max_ difference between the affected and normal adrenal gland of 1.0 (AUC = 0.992). The combination of both variables increased the AUC to 0.995.

**Conclusions:**

^18^F-FDOPA PET/CT distinguishes pheochromocytoma from normal adrenal glands with the highest diagnostic accuracy when combining the SUVmax of the affected adrenal gland with the difference in SUV_max_ between affected and normal adrenal gland.

## Introduction

Pheochromocytoma are rare neuroendocrine tumours originating from chromaffin tissue in the adrenal medulla, which demonstrate hypersecretion of catecholamines [1, 2]. Patients with pheochromocytoma characteristically have episodes of increased plasma catecholamine levels, which cause symptoms such as headaches, palpitations, anxiety and diaphoresis. Furthermore, paroxysmal or persistent hypertension, may occur [1]. The introduction of 6-[18F]-L-fluoro-L-3, 4-dihydroxyphenylalanine (^18^F-FDOPA) positron emission tomography with complementary computed tomography (PET/CT) provided a new approach of detecting and visualizing neuroendocrine tumours, compared to metaiodobenzylguanidine scintigraphy [3, 4]. ^18^F-FDOPA, in structure similar to L-DOPA, is the precursor of catecholamines [5]. It enters neuroendocrine cells by the large amino acid transporter (LAT1/CD98) and is subsequently converted by aromatic L-amino acid decarboxylase in ^18^F-fluorodopamine and ^18^F-fluoronorepinephrine [5–7]. Several studies have demonstrated that ^18^F-FDOPA PET/CT is well suited for the localization and visualization of pheochromocytoma [3, 7, 8]. However, differentiation between pheochromocytoma and normal adrenal gland can be difficult, since there is a large variability in physiological ^18^F-FDOPA uptake by normal adrenal gland tissue. In addition, (non-secreting) pheochromocytoma may show only faint ^18^F-FDOPA accumulation [9].

Although European Association of Research for Life (EARL) based standardized uptake value (SUV) determinations is common practice in ^18^F labelled flourodesoxyglucose imaging, studies using EARL-based SUV measurements in ^18^F-FDOPA imaging are rather scarce. These EARL are considered helpful in comparing SUV measurements between different centres, both in clinical settings and in multicentre trials. As of yet, cut-off values for physiological ^18^F-FDOPA uptake in adrenal glands have not been established. In addition, EARL-based SUV measurements of ^18^F-FDOPA accumulation in neither normal adrenal glands nor pheochromocytoma have been defined.

Therefore, the aim of this study was to describe reference ranges for ^18^F-FDOPA uptake by normal adrenal glands, and to establish EARL-based cut-off values for ^18^F-FDOPA uptake which can be used to reliably discriminate between a pheochromocytoma and normal adrenal gland tissue.

## Subjects and methods

All patients who received a histological diagnosis of pheochromocytoma in our hospital between November 2009 and December 2017 were included in this retrospective study. ^18^F-FDOPA-PET/CT scans were acquired in all patients. In pheochromocytoma patients, only the ^18^F-FDOPA-PET/CT scan for localization purposes before adrenalectomy and for possible metastases was used for this retrospective analysis. In disease control subjects who underwent multiple ^18^F-FDOPA PET/CT scans, only the first ^18^F-FDOPA PET/CT scan was analysed.

The disease control group consisted of patients who underwent ^18^F-FDOPA PET/CT for other indications (including neuroendocrine (carcinoid) tumours, medullary thyroid carcinoma, hyperinsulinaemia, and pancreatic isletcell tumours). Disease control subjects were not actively screened for germline mutations, but excluded from this group in case of known genetic susceptibility for developing a pheochromocytoma. These subjects were retrieved from a previously collected database of patients who underwent ^18^F-FDOPA PET/CT scanning in our centre [10, 11]. This database contained 338 patients with two adrenal glands, who had received carbidopa pre-treatment before ^18^F-FDOPA PET/CT acquisition. Patients scanned because of the biochemical suspicion of harbouring a pheochromocytoma but in whom no pheochromocytoma was detected (*n* = 90), as well as carriers of germ-line mutations predisposing for the development of pheochromocytoma (*n* = 3), were excluded from the database, resulting in 245 patients constituting the disease control group. As previously reported, steal phenomenon was considered non-existing [11].

Additional laboratory test results of the biochemical analysis (i.e. plasma (nor)metanephrine levels) were retrieved from the electronic patient charts.

### ^18^F-FDOPA PET/CT scanning and analysis

All patients were pre-treated with carbidopa, and received a median dose of 150 mg (range 75–150 according to body weight). In all patients, a low dose CT scan was performed for attenuation correction and anatomical localization. All ^18^F-FDOPA PET/CT images were acquired from top of the skull through mid-thigh 60 ± 6 min after intravenous administration of a standard dose of 200 MBq ^18^F-FDOPA on a Biograph mCT camera (Siemens Medical Systems, Knoxville, TN, USA). All patients fasted for 6 h and were allowed to continue all medication. Acquisition was performed in seven bed positions of 2 min emission time for patients between 60 and 90 kg. Patients with a body weight less than 60 kg and more than 90 kg body weight were scanned with 1 min and 3 min per bed position, respectively. Raw data were reconstructed through ultra high definition (Siemens) and according to guideline-based standardized EARL algorithms [12, 13] for SUV calculations, respectively [10]. Primary interpretation during clinical assessment was not taken into account for the purpose of this retrospective study. The adrenal uptake of ^18^F-FDOPA was measured using manually drawn spherical volumes of interests (VOIs) on EARL reconstructed PET images, to analyze tracer uptake in the left and right adrenal gland, using Siemens Syngo.via software version VB10. Uptake was expressed as maximum standardized uptake values (SUV_max_). Adrenal lesions were excluded from further analysis if, for example, the liver masked the adrenal uptake values. Image analysis was performed by MS, supervised by experienced nuclear medicine physicians (WN and AG), with 10 and 15 years’ experience in reading ^18^F-FDOPA PET/CT scans, respectively. Readers were not blinded for clinical history or previous imaging findings.

### Laboratory analysis

Plasma free metanephrine levels were determined using high-pressure liquid chromatography tandem mass spectrometry (LC-MS/MS) with online solid-phase extraction [14]. Laboratory results were determined within 3 months before or 3 months after the ^18^F-FDOPA PET/CT scan, in our in-house laboratory facilities only.

### Statistical analysis

Results are expressed as median and ranges. Only nonparametric tests were used to compare variables between patient groups. Receiver operating characteristic (ROC) analysis was performed for adrenal gland SUV_max_, and intra-individual difference in SUV_max_ between affected and normal adrenal gland. The highest SUV_max_ of both adrenal glands was used. The patients with bilateral pheochromocytoma were counted twice for inter-individual analysis, and excluded for intra-individual analysis. In addition, binary logistic regression was performed for ROC analysis of the combined parameters. Statistical analysis was performed using the SPSS package version 23 (IBM). A two-sided *P* value <0.05 was considered significant.

### Ethical consideration

According to the Dutch Medical Research Involving Human Subject Act, the local medical ethical committee (i.e. Medical Ethics Review Board of the University Medical Center Groningen, METc UMCG, number 201800952) exempted approval without additional procedures. No additional informed consent was required. Patient information was anonymized before data analysis.

## Results

### Patient characteristics

Characteristics of the study population are presented in Table 1. In total, 47 histologically confirmed pheochromocytomas were diagnosed in 45 patients. Patients with pheochromocytoma were younger than disease controls, both for the entire population and in females only.

**Table 1:** Characteristics of control subjects and patients harbouring a pheochromocytoma

Characteristic		Controls (N = 245)	Patients with pheochromocytoma (N = 45)	p value
Gender	Female	132	24	NS
	Male	113	21	NS
Age (years)	Median (range) of total population	61 (20–82)	50 (11–77)	*P* = 0.005
	Female	61 (20–81)	53 (11–77)	*P* = 0.016
	Male	60 (20–82)	55 (27–80)	NS
Plasma Metanephrine (nmol/L)	Median (range)	N/A	1.2 (0.070–38)	N/A
Plasma Normetanephrine (nmol/L)	Median (range)	N/A	3.0 (0.58–78)	N/A

Data presented as absolute values, or median (range). *NS* non-significant, *N/A* not applicable

**Fig. 1:**
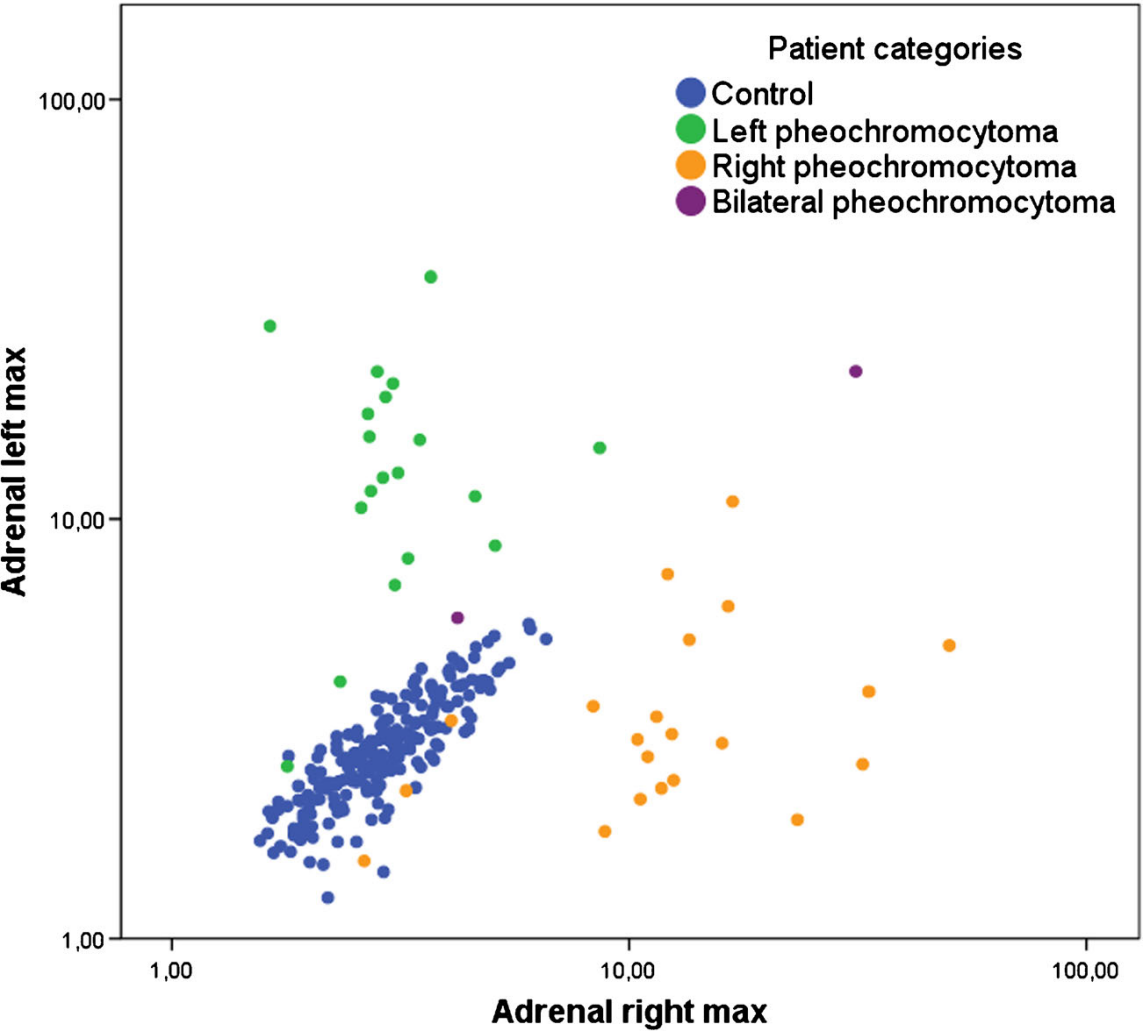
Comparison between the maximum standard uptake values (SUV_max_) of the right and left adrenal gland for the control group and the patients with pheochromocytoma (logarithmic scale)

Histopathologic analysis after adrenalectomy confirmed the presence of a pheochromocytoma in 45 patients, of which 24 were left sided and 23 were right sided (Fig. 1). Two patients had synchronous bilateral pheochromocytoma. Of these 45 patients, DNA analysis revealed germ-line mutations in the following genes: MEN2A in seven patients, SDHA in four patients, MAX in two patients, and NF-1 in one patient. In three patients, no DNA analysis was performed. In 28 patients, no germ-line mutation could be confirmed.

### ^18^F-FDOPA PET/CT findings

#### Patients with pheochromocytoma

Mean time between ^18^F-FDOPA PET/CT scan and adrenalectomy was 2 (0–6) months. Adrenal gland SUV_max_ of pheochromocytoma was higher than SUV_max_ of control adrenal glands: 12 (2.6–50) vs 2.9 (1.1–6.6), (*p* < 0.001). The difference in SUV_max_ of the pheochromocytoma compared to the contralateral normal adrenal gland was 14 (2.2–50). This difference in SUV_max_ was statistically different from the SUV_max_ difference between ipsilateral and contralateral adrenal gland in the control group (*p* < 0.001). No correlation was found between plasma (nor)metanephrines, and adrenal glands SUV_max_.

**Fig. 2:**
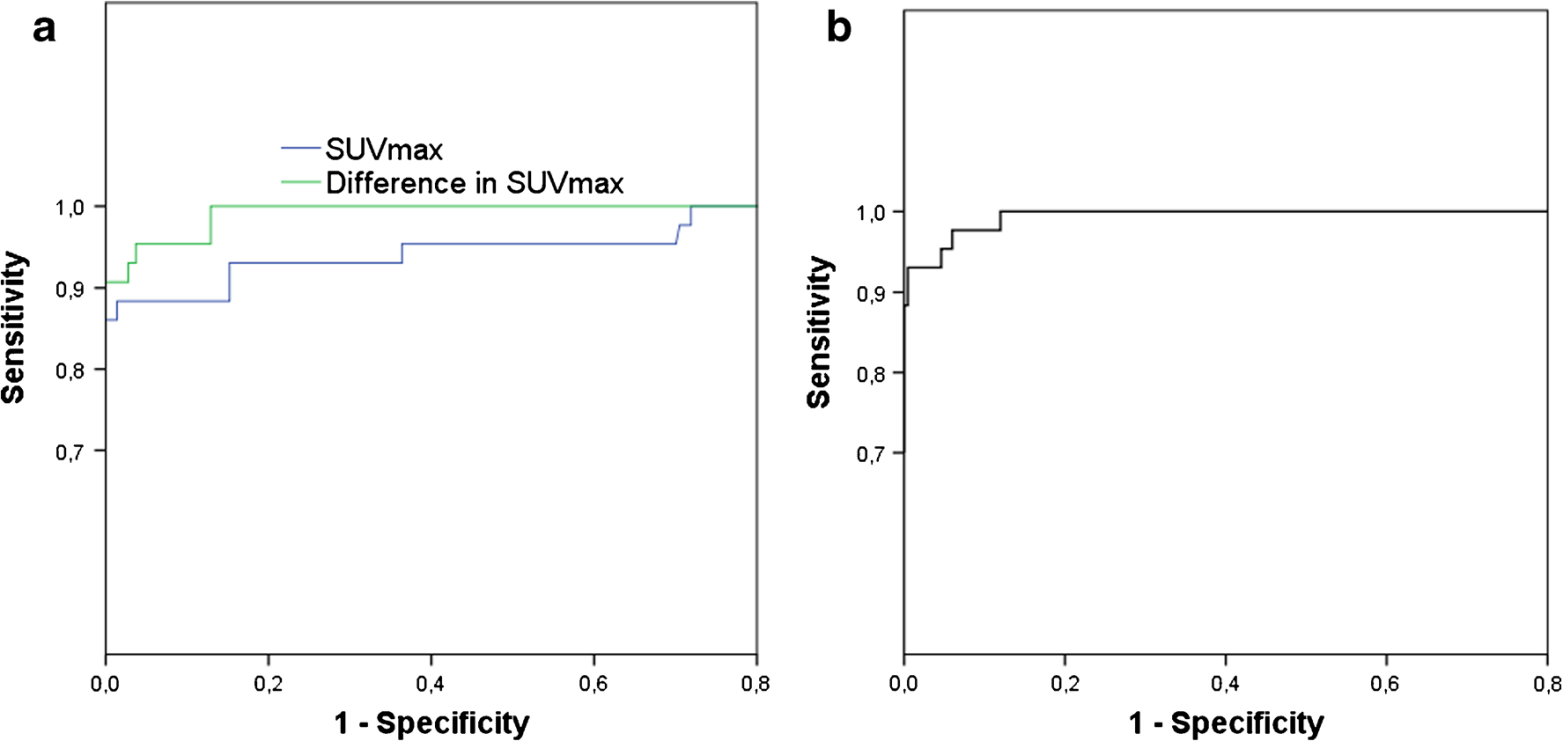
ROC curves for both adrenal gland SUV_max_ > 4.1, and intra-individual difference in adrenal gland SUV_max_ > 1.0 (**A**), and combined ROC curve (**B**)

**Fig. 3:**
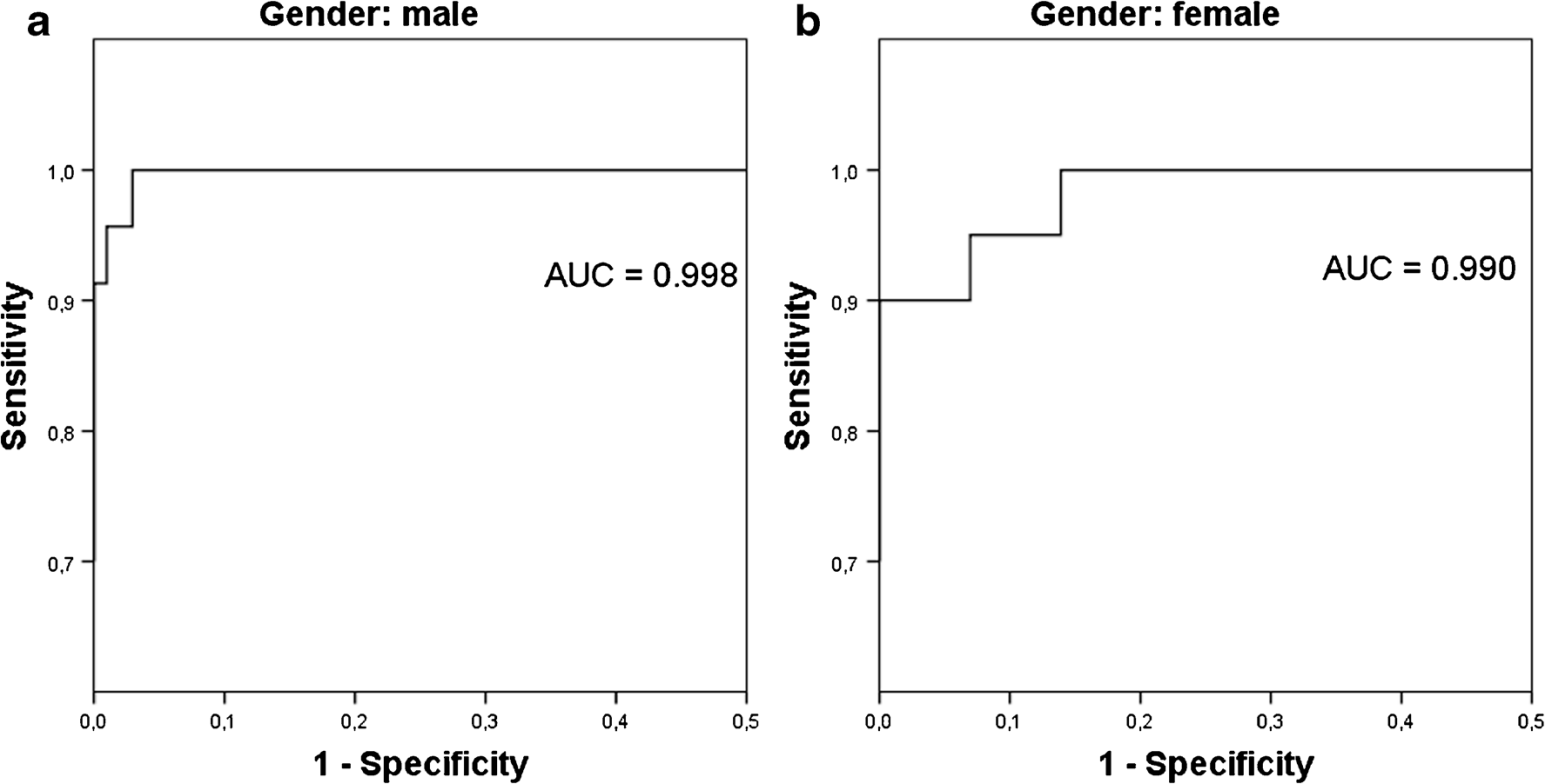
Combined ROC curve for male (**A**) and female (*B*) patients

Due to the overlap in SUV_max_ ranges between pheochromocytoma and disease control patients, receiver operating characteristic (ROC) analysis was used to determine cut-off values. The ROC analysis demonstrated that a cut-off SUV_max_ of 4.1 resulted in 93% sensitivity and 85% specificity for diagnosing pheochromocytoma, with an area under the curve (AUC) of 0.951 (Fig. 3A). In females a cut-off SUV_max_ of 4.0 resulted in 90% sensitivity and 80% specificity, whereas in males a cut-off SUV_max_ of 4.1 showed 96% sensitivity and 89% specificity. In addition, ROC analysis was performed to determine a cut-off value regarding the difference in SUV_max_ between the pheochromocytoma and the normal contralateral adrenal gland (Fig. 2A). An intra-individual difference in adrenal gland SUV_max_ of 1.0 resulted in 93% sensitivity and 96% specificity for the diagnosis of pheochromocytoma (AUC = 0.992), and 100% sensitivity and 97% specificity in males only. In females, an intra-individual difference of 0.78 resulted in 95% sensitivity, and 96% specificity. Using binary logistic regression for ROC analysis of the combined parameters increased the AUC to 0.995 (Fig. 2B), and 0.998 and 0.990 for males and females, respectively (Fig. 3).

#### Disease control group

**Fig. 4:**
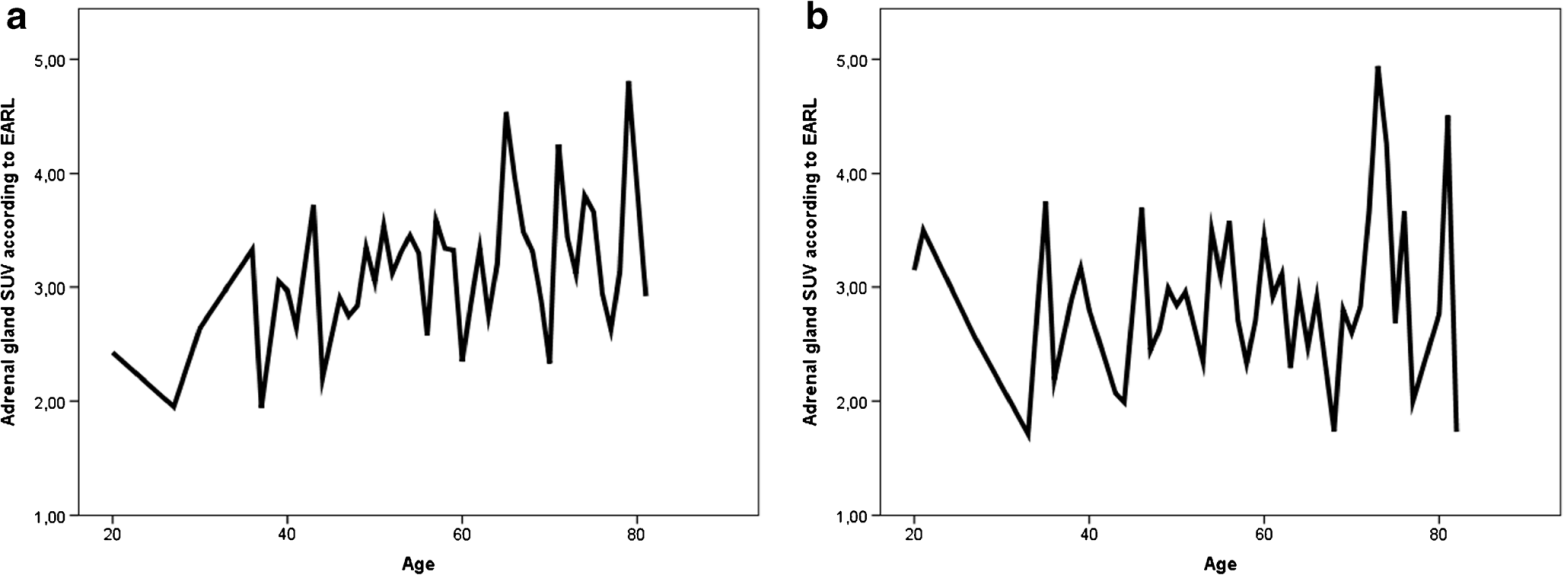
Line plots of mean standard uptake values (SUV_max_) in disease controls (**A** females, **B** males) per patient’s age, showing an age increase in SUV_max_ with increase in age in females only

Table 2 summarizes the SUV_max_ of the disease control group, which are considered normal reference values for future studies. The intra-individual (i.e. left versus right within the same subject) difference in SUV_max_ between the two adrenal glands was 0.30 (0.010–1.5). Within the whole group of control subjects, SUV_max_ of either one adrenal gland was significantly correlated to SUV_max_ of the contralateral adrenal gland (r = 0.86, *p* < 0.001, Fig. 1). This was consistent in male and female subjects only: r = 0.86 and r = 0.85, respectively, *p* < 0.001. However, adrenal gland SUV_max_ was higher in women than in men: 3.1 (1.8–6.6) and 2.8 (1.1–6.1), respectively (*p* < 0.001). In addition, only in women a statistical significant correlation between SUV_max_ and increase in age was found (*p* = 0.024) (Fig. 4).

**Table 2:** Comparison of ^18^F-FDOPA standardized uptake values (SUV_max_) in left and right adrenal glands in control groups, with *p* values

Measure	Controls			p value
	Total (N = 245)	Males (N = 113)	Females (N = 132)	
Left SUV_max_	2.8 (1.1–5.6)	2.6 (1.1–5.5)	3.0 (1.5–5.6)	*p* < 0.001
Right SUV_max_	2.9 (1.6–6.6)	2.7 (1.6–6.1)	3.0 (1.6–6.6)	*p* < 0.001
Intra-individual difference	0.30 (0.010–1.5)	0.30 (0.020–1.5)	0.30 (0.010–1.4)	NS
Correlation coefficient (Spearman’s rho)	0.86	0.85	0.86	*p* < 0.001

## Discussion

This is the first study to compare EARL based ^18^F-FDOPA accumulation in a large group of histologically confirmed pheochromocytoma to a large cohort of disease control patients, resulting in reference values for future studies. Based on this retrospective analysis, both inter- and intra-individual adrenal SUV’s on ^18^F-FDOPA PET/CT show very high test characteristics in distinguishing pheochromocytoma from normal adrenal glands, with an intra-individual difference in SUV of 1.0 showing the highest AUC. Furthermore, it appears that males and females show different normal adrenal gland SUVs, allowing different inter- and intra-individual cut-off values for identifying pheochromocytoma.

Correct localization of pheochromocytoma is of clinical importance, since adrenalectomy is the only curative treatment. For that purpose, reference values for normal ^18^F-FDOPA uptake are pivotal in accurately discriminating normal adrenal glands from pheochromocytoma. Several reports have shown that test characteristics of ^18^F-FDOPA PET/CT, ^18^F-FDOPA PET alone, and iodine-123 labelled meta-iodobenzylguanidine scintigraphy are not significantly different in the identification of pheochromocytoma [15, 16]. Previous studies reported high test characteristics for ^18^F-FDOPA PET/CT in the identification of pheochromocytoma, with 84–100% sensitivity and 88–100% specificity [16–18]. However, normal values of ^18^F-FDOPA uptake have not yet been retrieved from large groups. The most recent study that tried to provide more insight in both normal adrenal gland and pheochromocytoma ^18^F-FDOPA accumulations, retrospectively analysed 112 ^18^F-FDOPA PET scans [17]. The authors re-assessed 212 adrenal glands, of which 17 were pheochromocytoma (with histology confirmation in six cases). However, neither EARL-based ^18^F-FDOPA cut-off SUV_max_ for pheochromocytoma, nor EARL-based normal adrenal gland ^18^F-FDOPA SUV_max_ have been reported for males and females, thus far.

Currently, the main role of ^18^F-FDOPA PET/CT in pheochromocytoma is in localization of the tumour. Future prospective studies are needed to further explore the value of the presented ^18^F-FDOPA cut-off SUV in patients with a biochemical suspicion of a pheochromocytoma. Additionally, in this retrospective study only adrenal glands from patients with pheochromocytoma and disease control patients were re-assessed. As of yet, little is known of SUV_max_ in adrenal medullary hyperplasia. The value of ^18^F-FDOPA PET/CT in distinguishing adrenal hyperplasia from normal adrenal tissue has to be investigated in future studies.

### Limitations

In addition to the retrospective character of this study, potentially resulting in selection bias, there are some limitations of this study. Although this study contains a rather large group of pheochromocytoma patients, and a large group of disease control subjects, no power analysis has been performed to solidify the test characteristics.

SUVs of other organs than the adrenal glands have not been re-assessed in this study. However, female control subjects showing higher SUVs than male control subjects is in line with findings from a previous study from our group, in which we studied patients with post gastric bypass surgery hypoglcemia [11]. We are not yet aware of any other organ displaying a difference in SUV_max_ between males and females. As of yet, the clinical relevance of a significant difference between male and female adrenal gland SUV_max_, with higher values in women than in men remains to be determined.

Urinary (nor)metanephrine levels were not retrieved from the patient charts. Recently, however, urinary metanephrine levels showed a strong correlation with total lesion uptake in 56 patients with non-metastatic pheochromocytoma [18]. At present it is unclear if the difference of plasma versus urinary (nor)metanephrine levels is sufficient to explain the difference in correlation.

## Conclusion

^18^F-FDOPA PET/CT accurately distinguishes pheochromocytoma from normal adrenal glands, with the highest diagnostic accuracy when combining the SUV_max_ of the affected adrenal gland with the difference in SUV_max_ between affected and normal adrenal gland. In addition, in control subjects, women showed higher adrenal gland ^18^F-FDOPA accumulation than men. Therefore, we propose different cut-off SUV_max_ for women and men.
